# Application of relational tool to support acute cardiac patients’ emotional recovery: analysis of effects during acute and follow-up care

**DOI:** 10.1080/17482631.2019.1595957

**Published:** 2019-04-19

**Authors:** Daniela Berardo, Maria Valentina Mussa

**Affiliations:** Faculty of Nursing, Department of Public Health and Pediatrics Sciences, University of Turin, Turin, Italy

**Keywords:** Emotional recovery, relational tool, coronary heart disease, anxiety; quality of life

## Abstract

After an acute cardiac event, many patients experience emotional disturbance. This is a normal response to the event and to hospitalization, but, if not treated with emotive and social support, the symptoms can evolve, resulting in emotional and behavioural disorders. The aim of the study was to evaluate the outcome of the use of a new nursing relational tool, designed to support patients’ emotional recovery. The data are the result of semi-structured narrative interviews, conducted in the intensive cardio care unit of the Mauriziano hospital of Turin and by telephone, during March and August of 2017. The research sample involved 26 patients, divided into control and intervention groups. Exclusion criteria were: under age of 18, cognitive impairment or dementia, difficulty with comprehension and expression in Italian, and previous acute cardiac events. The interviews have shown that an emotional management tool improves the patient’s recovery, the quality of information received, and the quality of the patient’s everyday life after the event. The use of a relational tool by nursing staff improves the patient’s emotional management and increases the knowledge required to ensure a good quality of life. Continuous use also improves the confidence of health professionals in managing these emotional conditions.

## Introduction

The economic and social weight of hospitalizations resulting from cardiovascular diseases is increasing; more than half of these hospitalizations are due to chronic evolutions and complications from acute events. Patients who survive acute events often must deal with chronic conditions that affect their quality of life and that come with economic and social costs (Alleanza Italiana per le Malattie Cardio-Cerebrovascolari, 2017).

**Table I.** Control group.

Patient ID reference	Age (years/gender)	Diagnosis
PC1	29/M	Chest pain and non-sustained VT
PC2	54/M	STEMI
PC3	47/M	STEMI
PC4	76/F	N-STEMI
PC5	55/M	STEMI
PC6	68/M	STEMI
PC7	49/F	N-STEMI
PC8	66/F	STEMI
PC9	55/M	STEMI
PC10	68/M	STEMI
PC11	76/M	Angina pectoris and STEMI
PC12	66/M	STEMI
PC13	54/F	STEMI
PC14	73/M	N-STEMI
PC15	53/M	STEMI

**Table II.** Intervention group.

Patient ID reference	Age (years/gender)	Diagnosis
PI1	88/M	Syncope and type III A-V block
PI2	82/F	STEMI
PI3	73/M	STEMI
PI4	49/M	N-STEMI
PI5	50/M	STEMI
PI6	68/M	STEMI
PI7	83/M	N-STEMI
PI8	55/M	STEMI
PI9	72/M	STEMI
PI10	59/F	STEMI
PI11	75/F	STEMI

**Table III.** Thematic categories and descriptive units of control group.

Thematic categories	Descriptive units
1. The heart disease and its onset	1.1 Recognition of signs and symptoms 1.2 The emotive experience
2. Hospitalization	2.1 Awareness of the severity of the event 2.2 Personal response to the event
3. Perception of the disease	3.1 Uncertainty about the clinical evolution of the pathology and its duration 3.2 Knowledge of risk factors 3.3 Need to make behavioural changes 3.4 Need to understand own clinical condition 3.5 Disbelief at own illness
4. Relationship with the nursing staff	4.1 Care 4.2 Communication and sense of hospitality
5. Return to everyday life	5.1 Fear and concern for the return to everyday life 5.2 Fear of disability 5.3 Changes to everyday life

**Table IV.** Thematic categories and descriptive units of intervention group.

Thematic categories	Descriptive units
1. The heart disease and its onset	1.1 Recognition of signs and symptoms 1.2 The emotive experience
2. Hospitalization	2.1 Awareness of the severity of the event 2.2 Personal response to the event
3. Perception of the disease	3.1 Knowledge about the clinical evolution of the pathology and its duration 3.2 Knowledge of risk factors 3.3 Need to make behavioural changes 3.4 Satisfactory knowledge of own clinical condition 3.5 Disbelief at own illness
4. Relationship with the nursing staff	4.1 Care 4.2 Emotional support 4.3 Revaluation of nursing professional figure
5. Return to everyday life	5.1 Fear and concern for the return to everyday life 5.2 Fear of disability 5.3 Changes to everyday life

Acute cardiac patients can experience different emotions, like shock, low or fluctuating moods, sadness, worry, guilt, and anger; mood changes are displayed by tiredness, irritability, tearfulness, loss of pleasure in usual activities, withdrawal from others, sleep disturbance, and changes in appetite and sex drive. There are also disturbances of the social field, like reduced self-esteem, concerns about role changes, and pessimism about the future. This distress is considered a normal response to loss and to increased awareness of mortality; frequently, it has a spontaneous resolution within a few months, but some patients present a negative evolution of these conditions, developing anxiety disorders and major depression (Murphy, Higgins, & Jackson, 2015).

People who suffer from a cardiovascular event often do not receive adequate information about care continuation, rehabilitation, the chronic condition that affects them, and the need to change their eating and behavioural habits to prevent complications and to improve their quality of life (Pryor, Page, Patsamanis, & Jolly, 2014).

Currently, health professionals have basic notions on the principles of the helping and human relations. The most specialized training, as a counsellor, is the result of personal interest and not part of basic training. For health professionals, the emotional recovery of the cardiac patient has to be considered a central care element. Personalizing the treatment and making the patient really part of the care process is necessary to encourage a conscious change. To address this need, relational tools have been developed, for nurses, with the purpose of leading the patient towards a better quality of life and supporting the patient in this moment of fragility. Furthermore, such a tool, when shared by the whole team, guarantees uniformity of care and simplifies the insertion of new professionals in the team when necessary (Murphy et al., 2016).

The aim of the study was to evaluate the effectiveness of a new nursing relational tool for the emotional support of the acute cardiac patients. In particular, the tool is examined for efficacy in reducing the patient’s levels of anxiety, improving the patient’s awareness of the risk of mortality, helping the patient to understand the personal clinical condition, and encouraging the patient to explore resources to implement changes.

## Methods

### Processing of the relational tool

The project began in September 2016, engaging the nurses working in the intensive cardio care unit of the Mauriziano hospital in Turin. These nurses participated in a training course about effective communication. From this training and from analysis of the extant literature, a relational tool was developed addressing both the patients’ hospitalization and their time after discharge.

The tool encompasses consideration of concepts like self-efficacy (Bandura, 1986) and illness belief (Lau-Walker, 2006, 2007); communication techniques were introduced to improve active listening, self-exploration, and the active involvement of the person (Lau-Walker, Landy, & Murrells, 2016). The tool is composed of four general steps: meeting, comprehension, information, and planning. These steps are elaborated in 14 more detailed steps. The tool is composed of easy, general rules, based on principles of helping relation (Carkhuff, 1994) and effective communication techniques (Table in Appendix).

### Design and setting

The study provided for the evaluation of the effectiveness of this tool through qualitative interviews.

The semi-structured interviews were conducted in the Mauriziano hospital of Turin, in the intensive cardio care unit. The sample was of 26 patients, divided into, first, a control group, who received the standard professional treatment, and, second, an intervention group, who received a structured treatment implementing the new relational tool. Semi-structured interviews were conducted in person at the time of initial hospitalization after an acute event; 2 to 3 months later, follow-up interviews were conducted by telephone.

### Participants

The inclusion criteria selected for patients suffering, for the first time, an acute cardio disease, either hemodynamic or arrhythmological. The exclusion criteria were: under age of 18, the presence of cognitive deficits or dementia, difficulty in comprehending or communicating in the Italian language, and experience of a previous acute cardiac event.

Eligible patients were identified by nursing staff; the researcher explained the study and asked for patients’ consent and their availability for contact by telephone in the following months.

The control group was formed by 15 patients, aged from 29 to 76, 11 male and 4 female (Table I).

The intervention group was formed by 11 patients, aged from 49 to 88, 8 male and 3 female (Table II).

### Data collection

Data were collected with qualitative interviews, which were conducted and recorded during the hospitalization and in an undisturbed place. During the interviews the researchers and the respondents talked in Italian language. All the interviews were conducted following a predetermined set of questions, they started with an open question: “Can you tell me what happened and how you felt?” This was followed by explorative questions to investigate the patient’s emotional state like “Did you have worrying thoughts?” and “Did someone help you to explore your concerns?,” previous and developed knowledge, and expectations for the future like “How much control do you feel you have over your illness?” The questions were influenced by the Hospital Anxiety and Depression Scale (Hunt-Shanks, Blanchard, Reid, Fortier, & Cappelli, 2010; Zigmond & Snaith, 1983) and The Brief Illness Perception Questionnaire (Broadbent, Petrie, Main, & Weinman, 2006) and examining the needs declared in previous studies related to the topic. (Svedlund, Danielson, & Norberg, 2001; Wieslander, Martensson, Fridlund, & Svedberg, 2016). The interviews lasted between 8 and 30 minutes; the interviewer dialogued with the patients interrupting them as little as possible and briefly reformulating the less clear passages, allowing the patient-free expression. All the interviews were recorded and transcribed to ensure the best possible analysis.

To analyse the follow-up effects, interviews were conducted by telephone, using short and closed questions. These question/answer sets were inspired by the Morisky Medication Adherence Scale (Morisky, Ang, Krousel-Wood, & Ward, 2008), the Dartmouth COOP Quality of Life Questionnaire (Jenkinson, Mayou, Day, Garratt, & Juszczak, 2002) and SF-36 (Apolone & Mosconi, 1998).

### Ethical consideration

The Mauriziano Hospital approved the study, authorizing access to elements of the patients’ data and treatments and collaboration with hospital personnel. Informed consent was obtained after the participants were fully informed, through a summary written letter and verbal discussions, about the aim of the study, the use of their personal data, the voluntary nature of participation, and their ability to withdraw.

### Data analysis

Data were processed by the authors, individually, to prevent personal bias, using Giorgi’s method (Giorgi, 2009). The interviews were read through several times; after a researchers’ comparison, they are subdivided into thematic categories and descriptive units (Table III and IV). The answers to the follow-up questionnaire were collected and evaluated. At the end of the analysis, the two groups were compared to evaluate the differences and to detect the effectiveness of the intervention. The analysis focused on identifying the effects induced by the relational tool and not shown by the control group.

## Findings

### Control group

#### The heart disease and its onset

In the description of the event, the majority of patients recalled fairly typical symptomatology. Many of them perceived that their experience was a result of cardiac disease. Some undervalued the signs, but all of them believed hospital exams to be necessary. PC13 offered a typical response: “Suddenly, I felt a pain in my chest and back, I felt prickles in the legs and arms, I began to vomit and lose consciousness. I lost consciousness, and I had a terrible pain.”

The patients’ emotional experiences were also very important, to them and to this study. In addition to physical pain, patients described disturbing emotional states arising. They reported experiencing an imminent sense of death and negative thoughts related to loved ones and things that would be left unfinished, as PC2 reported: “The peak was very bad, very scary. I quietly thought not to see the next day.” Some of the patients felt confused; they felt they could not really understand what was happening and had difficulty cooperating with those offering aid, because of their confusion.

#### Hospitalization

After the diagnosis, many patients realized the risks they have taken and the impact on their lives. In a short time, from a situation of apparent well-being, they moved instead into patients suffering from a serious illness that will make significant changes to their daily lives.

During the hospitalization, the patients’ personal responses were varied and not easy to understand. Often, they claimed to have already overcome the event, but they were reluctant to deepen the emotional state, giving ambiguous and impulsive answers. PC6, for example, articulated that “I can confess—they don’t have to tell me there is a God, because I am not an atheist—but prayers do not help. ”

#### Perception of the disease

There is uncertainty about the course of the disease; many patients did not understand the influence that their diagnosis will have in everyday life. Furthermore, different patients expressed quite different ideas about the duration of the disease. PC12 gave the response “Ten years ago it would have been a heart disease, now it’s a problem solved.” As shown by this belief that the problem is “solved,” the patients’ knowledge of risk factors is doubtful, and their limited knowledge may be the result of knowledge disseminated through primary prevention or given by family experience.

Almost all the interviewees understood the need for some behavioural changes, but the indications, from hospital staff, of what such changes should encompass were not sufficiently clear, leaving the patient at the mercy of personal beliefs and previous knowledge. Only a small part believed that changes will not be necessary. PC4 expressed both uncertainty and a willingness to adopt change, saying “Well, I do not know; if I have to pay more attention, then they will tell me.” There was a different consciousness in the group about the follow-up care, but they did not know what they have to do.

I will have to make choices about a certain kind of life that I have had so far—choices that will cost me a lot, because they are things that I like to do and that I will not be able to do anymore, but I am optimistic. (PC7)

Many patients expressed the need to learn more about their clinical conditions; they reported that they have not received enough information and believe that more information should be given by the doctor. PC8 offered a typical response of this sort, saying “It is not clear; I would like to understand more, how this happened. I’m just waiting for the doctor to explain to me what I should do.”

A small part of the group did not understand why they were affected by this pathology, despite knowing some of the risk factors.

#### Relationship with the nursing staff

Patients’ experiences with the hospital staff were good; the staff appeared to be competent and helpful. Professionalism was mainly expressed in the technical practice and not in the relational one. PC9 expressed well this concepts: “Well, here, they care; it’s not that they ask you..there’s no one like you that wants to go a little deeper. We’re all the same: they cure you, they attach you on this stuff, these machines.”

Some patients reported a good sense of hospitality and generally felt they have received good support. Others reported that they were not satisfied with the service they received or that they felt convinced that relational considerations were not important to those in hospital staff professions.

#### Return to everyday life

The patients reported a fear of losing loved ones as encouraging them to work for change in their lives. Only a small group reported a fear of being disabled, or of other risks that might affect their quality of life. Most believed that the incident will not affect their lives in the long-term; the attention on their lifestyle will be increased for only a few months, they believed, and then they will return to complete normalcy. “I saw so many other people who did the same thing and now do the same things as before, so I do not see why it should be different for me” (PC12).

### Intervention group

#### The heart disease and its onset

There are not a lot of differences between the two groups in their responses to the acute event; the patients’ rapid recognition of signs and symptoms was quite evident. In terms of emotional response, fear was the main emotion experienced.

#### Hospitalization

The acquisition of awareness of the severity of the event was similar to the first group. The response to the event was very personal. “This was an alert, a warning, because I think we need to give ourselves a cut. I have to start thinking about being a little quieter, not to run so much” (PI11).

#### Perception of the disease

The clinical knowledge of patients in the intervention group was better than that of patients in the control group; there is an awareness that the process they are beginning will not be short and will involve limitations. An example of this, was PI6’s answer: “I think they are quite long courses, they all say that it takes a lot of patience. Now I do not feel very bad, just a little limited.”

The knowledge of risk factors among the intervention group was similar to the knowledge of the control group; the reflection with the professional has, sometimes, led intervention group patients to greater consideration of some negative elements. The intervention group seemed to have a more realistic view of the clinical course, overall. Intervention group patients understood that the process will involve changes to their daily lives and that it will not be short or easy. PI6 showed a typical intervention group understanding:

There must be a series of guidelines to be respected, of things that perhaps can no longer be done, eaten, drank. There is all the aspect of things to do physically, how to move, what limits.. this is another thing that gives you the conception of your limits, in the early days you realise that certain things you cannot do, you do not have more breath to do them, and then you have limits.

In the intervention group, almost all patients felt they received clear and satisfactory answers on their clinical condition. Only a small part of the group, the oldest one, reported a personal lack of interest in the details, because the family has been informed of all.

Compared to the control group, there was a greater acceptance of the event and an increased sense of possession of better information.

#### Relationship with the nursing staff

The assistance provided was of good quality, and both patients and their families were very satisfied with the service. PI10 offered effusive praise in this area, saying “They were very nice here, above [in UTIC-intensive care unit] really, very nice, very sensitive, very welcoming. (In UTIC) very well, welcoming staff; there is a little difference compared to here (cardio ward), they explained everything to me, really exquisite people.”

Thanks to the relational intervention, patients reported having received good emotional support, being able to rework their experiences, and feeling welcomed and understood, respecting their own times.

PI9: They were very exhaustive, both this morning the doctor and today the nurse came to explain everything to me, to wonder if I realized what had happened, how and why. Researcher: it was useful?PI9: Yes, yes, very much, so I have a good vision.

In addition to the emotional support, for the patients, the professional nursing figures stood out for their knowledge and for their care performance. PI6 expressed the patients’ admiration for the nurses:

I would say that they have also helped all the people above [UTIC], they were very available also to .. not only in the aspect of care, which is fundamental, but also in some way in giving a support, even indirectly.

#### Return to everyday life

The return to everyday life brought different fears for different patients, but these fears were mainly related to their ability to resume their usual activities. Only a few patients had the fear of remaining disabled.

Thanks to the relational tool, that improved the communication; they were aware of the need to make changes to their lifestyles. However, as such changes were the result of a shared, rather than an imposed, choice, this necessity did not scare the patients. PI4 expressed this concept: “If we talk about illness, we talk about a different route, a condition, a different mental attitude in front of the roast pork, the cocktail, the wine, the cigarette” (PI4).

## Analysis of data collected at follow-up

Regarding adherence to the prescribed drug regimen, both groups followed the medical indications and respected the time and methods of consumption. Only smokers in the intervention group openly declared that they had quit smoking; smokers in the control group did not address any changes in their habits. Most patients reported they have changed their eating habits: they tended to reduce fatty foods and salt use. Only some cases reported that they have not made any changes. Regarding physical activity, no one reported having engaged in structured activities: most reported that they could not work out due to the physical conditions that arose, some say they could not due to the lack of time. Only three cases from the intervention group reported that they have changed their habits to include more frequent physical activity.

Emotions like anxiety, irritability, and sadness were present in all patients in the control group. Many of them reported not having been informed of the difficulty of recovery, causing dissatisfaction and anger. Others in the same group reported having received information that did not match their reality. In the intervention group, the main concern was the drug therapy: the difficulty of finding the therapy in case of travel, the large amount of drugs, and the need to be precise in the consumption. There was a moderate fear of not being able to fully resume their work activities, and of the associated consequences on their quality of life.

## Discussion

The cardiac rehabilitation was based mainly on the secondary prevention: preventing disease progression, reducing risk factors and mortality, by acting on the lifestyle. Recent studies, like this one, showed the importance of the behavioural, social and psychological dimensions for the cardiac patients. In this moment of fragility, emotional support is a patient need. For this reason, a valid relational support must be considered a priority equal to that of more technical intervention (Lau-Walker, Cowie, & Roughton, 2008). During the recovery, they have to find their inner strength and to develop self-efficacy in order to increase their well-being.

From the results, the intervention was positive; the same subjects declared their usefulness. As already highlighted in literature, the verbalization of real or perceived threats has helped to reduce anxiety and to promote the continuity of care. From the comparison of the two groups, it was noticed that those who did not receive the treatment had difficulty at the time of the narrative interview in recounting the event, often getting carried away by negative feelings. This difficulty was probably the result of a failure to rework the event. Moreover, the intervention group reported and reflected better education and preparation. In fact, the interviewees declared that their returns home were less tiring, because unexpected situations did not arise. The opposite happened in the control group, where information was misinterpreted or never provided, creating a lot of inconvenience for the people involved. Early education provided tools that help the patients to make changes easily. Many members of the control group did not consider changes to their lifestyle necessary, putting their health at further risk.

The study highlights the need for the nurse to work actively with the psychological and social dimensions in order to guarantee a good recovery process and a better quality of life.

The effective use of this tool undermines the concept of the passive patient to restore dignity, enhance patient resources, and improve the process of healing or cohabitation with the pathology. During the intervention, the nurse can accompany patients to find the necessary resources to overcome any declared discomfort and to face the chronic nature of the condition with more serenity. The professional aim should be not only to ensure quality care, but also, by exploiting the relational potential inherent in healthcare, to actively involve patients in their care plans. The use of a structured tool, such as the one proposed, can act as a guide to allow a professional change, with the purpose that each nurse acquires familiarity with the tool, personalizes the tool, and increases his or her personal skills.

## Conclusion

To respond to the needs of the patients implies a greater commitment for the health personal, but an adequate response considerably reduces the distress that could arise later. One importance consideration emerging from this study is the patient’s re-evaluation of the nurse: the members of the intervention group reveal amazement at the nurses’ knowledge, and they distinguish the nursing figure from the medical one and appreciate the exclusive time dedicated to them. Indeed, the nurse is the most suitable professional figure to participate in this intimate moment of fragility, is the only one able to establish a stable relationship of trust. For this reason the tool is directed to this professional figure.

## Appendix Appendix

### Hospitalization

Meeting with the patient and assessment.

Time: 15-20 minutes

**Table:**

**MEETING** Create favourable conditions to dialogue	1- Present yourself by name and profession 2- Assume a position at the same level as the person (symmetrical relationship) 3- Contact the person or refer to her avoiding appellations as “patient, sick, …”	**Confidence**
**COMPREHENSION** Demonstrate to understand the emotional condition	4-Promote self-exploration and leave space for the person, respecting the silence (active listening) 5- Return the person to the “here and now", keeping the focus on the current condition *“What do you feel now?", “What emotionally feels now?", “What would you like now?”* 6- Reassure the assisted person that the emotions felt are frequent and demonstrate to understand them. Do not banalize! *“The situation that has lived has frightened her a lot and now she fears she can get back, so she does not want to be alone”*	**Empathy** **Feeling** **Emotion** **Desire**
**INFORMATION** Inform on the clinical condition and the follow-up to reduce anxiety	7- Verify the clinical and anatomical knowledge of the person *“Did you understand what happened?", “Why did it happen, in your opinion?"*(investigate risk factors) 8- Strengthen the right beliefs and deny the wrong ones 9- Explain with simple language and using metaphors related to the world of the assisted person (his work, his hobbies), not taking anything for granted 10- Explain and motivate all nursing interventions on the person	**Illness Belief** **Suspension of judgment**
**PLANNING** Share the treatment plan actively involving the assisted person, to have good compliance	11- Explore the expectations of the person “*What do you imagine is happening?",“What do you expect to happen?", “How do you plan to tackle this event?”* 12- Explain what the diagnostic-therapeutic plan will be 13- Allow the person to ask questions 14- Search for feedback and verify consent	**Imagination** **Thought** **Intuition** **Self-efficacy**

### Discharge

Preparation of the assisted person and transfer to the ward.

Time: 15-20 minutes

**Table:**

**MEETING** Create favourable conditions to dialogue	1- Greeting the person 2- Assume a position at the same level as the person (symmetrical relationship) 3- Inform that now it will be necessary to change your habits and that the transfer to the ward will soon be carried out	**Confidence**
**COMPREHENSION** Demonstrate to understand their concerns and identify starting points for developing change	4- Explore the concerns, respecting the silence (active listening) 5- Demonstrate understanding of concerns using comprehensive reformulation “*Dependending on your wife for meals you fears you cannot follow the food instructions given to you”* 6- Explore the personal perception of illness (signs and symptoms, causes, chronic condition, physical, social, economic and emotional consequences, care and control)	**Listen** **Feeling** **Emotion** **Desire**
**INFORMATION** Inform on how to live with this pathology so that they accept the consequences	7- Strengthen the correct beliefs and alert to potential risks and how to recognize them (do not prohibition, but express the potential) 8- Declare that your active involvement improves the results 9- Emphasize how both the operator and the assisted person are responsible for managing health conditions	**Responsibility** **Awareness**
**PLANNING** Share the objectives to have good health management and to be autonomous	10- Identify with the person simple and close objectives to trigger changes 11- Explore the expectations of the person *“How do you imagine to change your eating habits?, What do you think you change?”* 12- Verify that the person has understood or evaluate if it is necessary to identify a method of learning that is more suitable for the person 13- Allow the person to ask questions 14- Greet and declare your availability for further clarification	**Imagination** **Thought** **Intuition** **Adherence** **Autonomy**

### General indications

**Table:**

Create favourable conditions to dialogue changing the setting
Ask the person if he/she wants a caregiver/relative close and who he/she prefers
Answer questions promptly, without shifting attention to other topics. Open a topic, conclude it without opening others
Avoid to use epithets (patient, sick, …) Avoid to use terms of endearment referring to nursing
Be aware that you cannot answer all the questions of the person
Leave space to the person and respect the silence
Maintain physical contact with the person
